# A Node-Adaptive Feature Fusion Network for Drug–Target Interaction Prediction Based on Multi-View Graphs

**DOI:** 10.3390/biom16060908

**Published:** 2026-06-18

**Authors:** Lin Xie, Hongmei Xu, Pinglu Zhang, Jianshe Xiong, Jing Li

**Affiliations:** 1 College of Electronic Engineering, Faculty of Information Science and Engineering, Ocean University of China, Qingdao 266404, China; xielin011223@stu.ouc.edu.cn (L.X.); tinaxu@ouc.edu.cn (H.X.); zpl1668@stu.ouc.edu.cn (P.Z.); 2 Key Laboratory of Marine Drugs, Chinese Ministry of Education, School of Medicine and Pharmacy, Ocean University of China, Qingdao 266003, China

**Keywords:** drug–target interaction prediction, multi-view graph, node-level adaptive fusion, graph representation learning

## Abstract

Existing drug–target interaction (DTI) prediction methods still face challenges caused by sparse interaction data, complex multi-source relationships, and imbalanced information contributions among different nodes. In this study, we propose NAFF-DTI, a node-level adaptive feature fusion network based on multi-view graphs. The model uniformly represents drug similarity, target similarity, and known drug–target interactions as multiple relational views, and learns node representations through graph encoding and cross-view representation learning. To more effectively utilize heterogeneous relational information, NAFF-DTI introduces cross-view feature discrepancy modeling and a node-level adaptive fusion mechanism to dynamically adjust the contribution of different views according to node structural characteristics. Experimental results show that NAFF-DTI achieves the best AUC and AUPR on all five benchmark datasets. Compared with the strongest baseline for each dataset and metric, NAFF-DTI achieves average relative improvements of 3.81% in AUC and 3.23% in AUPR. It can also improve the utilization of multi-source information, maintain relatively stable prediction under different data distributions, and prioritize biologically plausible candidate drug–target associations from the unannotated candidate space. These results indicate that NAFF-DTI can provide computational support for DTI candidate prioritization and repurposing-oriented hypothesis generation.

## 1. Introduction

Drug–target interaction (DTI) prediction is a key step in linking drug molecules with biological targets, and plays an important role in drug screening, mechanism investigation, and drug repurposing. Accurately identifying potential drug–target associations can help shorten the drug development cycle and reduce experimental costs. However, conventional experimental methods usually rely on costly and time-consuming biological assays, making it difficult to meet the demand for large-scale candidate drug screening. In this context, computational model-based DTI prediction has gradually become an important research direction [1]. In recent years, drug development has remained active, with the FDA Center for Drug Evaluation and Research (CDER) approving 46 novel drugs in 2025, further highlighting the continuing demand for efficient target identification, mechanism investigation, and computational DTI prediction support [2,3].

Existing DTI prediction methods have evolved from traditional feature modeling to network inference, and then to deep learning-based representation learning. Early studies mainly used ligand similarity, drug chemical structure similarity, prior drug–target features, or three-dimensional target structures for prediction and molecular docking analysis [4,5]. Subsequently, network-based methods modeled drug–target relationships as bipartite graphs or heterogeneous networks, and inferred potential interactions through network topology, random walks, or network diffusion [6,7]. These methods can capture high-order relational information, but their performance is usually affected by network completeness, propagation mechanisms, and existing connection patterns.

With the development of deep learning methods, DTI prediction has gradually shifted from approaches relying on hand-crafted features and fixed propagation rules to representation learning-based modeling. Debnath et al. [8] systematically reviewed deep learning-driven drug–target binding prediction studies, and pointed out that related methods have evolved from early heterogeneous network and sequence modeling to graph structure modeling, attention mechanisms, and multimodal fusion. They also summarized existing models in terms of commonly used datasets, evaluation metrics, and application cases. Sun et al. [9] proposed iNGNN-DTI, which combines a pretrained molecular model with nested graph neural networks to learn representations on drug- and protein-related graph structures, thereby enhancing the modeling of local structural information. Zhao et al. [10] proposed EviDTI based on evidential deep learning, integrating drug two-dimensional graphs, three-dimensional conformations, and target sequences into a unified framework to improve the predictive ability and reliability of DTI prediction. Qiao et al. [11] proposed CE-DTI, which models potential associations between drugs and targets through graph generation and multi-source information fusion, further demonstrates the importance of multi-source information fusion in DTI prediction. On this basis, Li et al. [12] proposed HGCML-DTI, which combines multi-view heterogeneous graph modeling with contrastive learning and learns drug and target node representations from different relational views, thereby improving the utilization of multi-source relational information. Liao et al. [13] proposed ContraDTI, which uses the molecular graph of a drug as the main view and the SMILES sequence as an auxiliary view. By introducing multi-view contrastive loss and cross-view alignment constraints, ContraDTI alleviates the influence of insufficient labeled data on model training, further showing the application value of contrastive learning in sparse DTI prediction scenarios.

The above studies show that deep learning methods have improved the representation learning ability of DTI prediction. However, existing methods still have limitations in multi-source relation modeling and feature fusion. Some methods cannot simultaneously characterize multi-source relationships such as drug similarity, target similarity, and known interactions. Existing multi-source fusion and multi-view contrastive learning methods usually integrate information at a global level, while insufficiently considering that different nodes may receive unequal contributions from different relational views. Related studies also indicate that data imbalance, insufficient cross-view information utilization, and limited model generalization remain important challenges in DTI prediction [14]. Therefore, how to achieve adaptive fusion by combining node structural characteristics with cross-view representation discrepancies is a key issue for improving the predictive performance and stability of DTI models.

To address these issues, we propose Node-level Adaptive Feature Fusion for Drug–Target Interaction Prediction (NAFF-DTI), a node-level adaptive feature fusion network based on multi-view graphs. NAFF-DTI is designed for the complex relational information, sparse interaction data, and imbalanced node-level information contributions in DTI prediction. It uniformly models drug similarity, target similarity, and known drug–target interactions as a multi-view graph structure, and extracts node features from different relational spaces during graph representation learning. On this basis, NAFF-DTI further models cross-view representation discrepancies and learns adaptive fusion weights by incorporating node structural information, allowing the model to dynamically adjust the feature fusion strategy according to the information status of different drugs or targets in each view. Compared with fixed or global fusion strategies, this approach can make fuller use of multi-source relational information and reduce the influence of redundant or noisy views on node representations, thereby improving the stability and discriminative ability of DTI prediction.

The main contributions of this paper are as follows:

- NAFF-DTI constructs a multi-view graph from drug similarity, target similarity, and known drug–target interactions, enabling node representation learning across multiple relational spaces and alleviating information insufficiency caused by sparse interactions.
- NAFF-DTI introduces cross-view feature discrepancy modeling and node-level adaptive fusion to capture the inconsistent contributions of different views and dynamically adjust fusion weights according to node structural characteristics.
- NAFF-DTI is systematically evaluated on multiple datasets through overall performance comparison, ablation experiments, hyperparameter sensitivity analysis, repeated holdout validation, cold-start and long-tail scenario analyses, and case studies.

## 2. Materials and Methods

To address the data sparsity and complex relational information in DTI prediction, we propose NAFF-DTI, a node-level adaptive feature fusion network based on multi-view graphs. In DTI data, drug similarity, target similarity, and known interactions describe potential drug–target associations from different perspectives. Therefore, relying on a single relationship type is insufficient for comprehensive representation learning. NAFF-DTI uniformly models these multi-source relationships as a multi-view graph structure and learns drug and target representations from different relational spaces.

**Figure 1:**
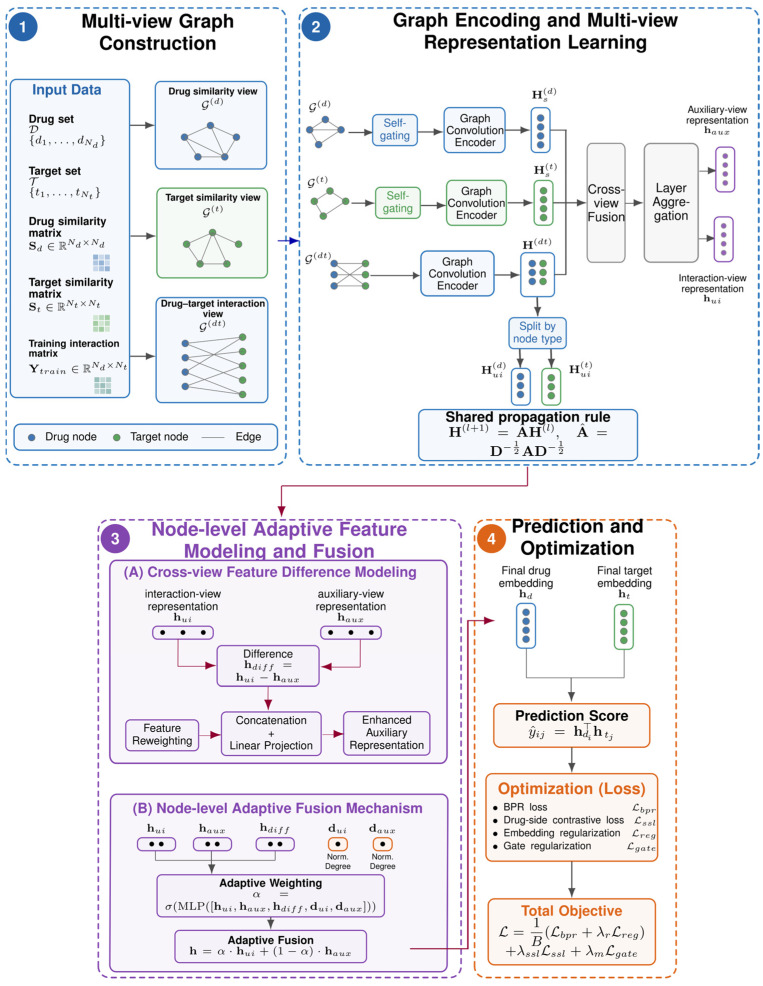
Overall framework of the proposed NAFF-DTI model.

As shown in Figure 1, NAFF-DTI consists of three main components: multi-view graph construction, graph encoding and multi-view representation learning, and node-level adaptive feature modeling and fusion. The prediction and optimization module is then used to calculate interaction scores and train the model. Specifically, NAFF-DTI first constructs graph views according to different relationships and performs representation learning on each view. It then integrates multi-view representations through aggregation and cross-view interaction. Finally, cross-view representation discrepancies and node structural degree information are used to learn adaptive fusion weights, enabling NAFF-DTI to adjust the fusion strategy for different nodes and provide stable representations for DTI prediction.

### 2.1. Problem Definition

In the DTI prediction task, the known drug set and target set are usually represented as two node sets. In this paper, targets mainly refer to protein targets, and this distinction is not repeated hereafter. Let the drug set be denoted as $D=\{d_{1},d_{2},\ldots ,d_{N_{d}}\}$ and the target set as $T=\{t_{1},t_{2},\ldots ,t_{N_{t}}\}$. The known interaction relationships can be represented as a bipartite graph or an adjacency matrix $Y\in R^{N_{d}\times N_{t}}$, where $Y_{ij}=1$ if drug $d_{i}$ and target $t_{j}$ have a verified interaction, and $Y_{ij}=0$ otherwise. It should be noted that, in existing DTI datasets, $Y_{ij}=0$ does not necessarily indicate that the corresponding drug–target pair has been experimentally verified as non-interacting, but usually means that no related evidence has been observed so far [15]. Following this common setting, we sample negative examples from unobserved drug–target pairs during model training, validation, and testing for model optimization, model selection, and performance evaluation. The goal of DTI prediction is to learn a function f:D×T→R based on the partially observed matrix Y, so as to estimate the probability or confidence of interactions between unknown drug–target pairs.

Different from traditional methods that only rely on the interaction matrix, practical DTI prediction is usually accompanied by multi-source auxiliary information, such as the drug similarity network $S_{d}\in R^{N_{d}\times N_{d}}$ and the target similarity network $S_{t}\in R^{N_{t}\times N_{t}}$. These types of information characterize potential association structures among drugs or targets from different perspectives, and can help alleviate the learning difficulty caused by sparse interaction data. Therefore, DTI prediction can be formulated as a multi-view graph-based data modeling problem. Specifically, node representations are jointly learned on multiple relational views ${\left\{G^{\left(v\right)}\right\}}_{v=1}^{V}$, and the fused representations are then used for interaction prediction.

For each type of relationship, such as drug–drug similarity, target–target similarity, and known drug–target interactions, a corresponding graph structure $G^{\left(v\right)}=(V^{\left(v\right)},E^{\left(v\right)})$ is constructed. Here, the node set $V^{\left(v\right)}$ contains drug or target nodes, and the edge set $E^{\left(v\right)}$ represents the relational connections under the corresponding view. Based on this formulation, the model needs to learn node embeddings $H^{\left(v\right)}$ in each view and obtain a unified representation H through an effective cross-view fusion strategy, so as to describe the comprehensive characteristics of nodes.

Therefore, the problem studied in this paper can be summarized as follows: given a multi-view graph structure and partially known interaction supervision signals, the aim is to design a representation learning method that can fully exploit different relational information and perform adaptive fusion, thereby improving the prediction ability for unknown drug–target relationships. This problem can be regarded as a combination of multi-view graph representation learning and bipartite graph link prediction. Its core challenge lies in how to achieve effective feature representation and fusion under sparse information and heterogeneous relationships.

### 2.2. Multi-View Graph Construction

Based on the above problem definition, we construct a multi-view graph structure to sufficiently characterize multi-source relational information between drugs and targets, and uniformly model different types of associations. Around the drug set D, the target set T, and the known interaction matrix Y, multiple relational views are introduced from two aspects: similarity relationships among homogeneous nodes and interaction relationships between heterogeneous nodes.

Based on the target similarity matrix $S_{t}\in R^{N_{t}\times N_{t}}$, the target similarity view is constructed as $G^{\left(t\right)}=(T,E^{\left(t\right)})$. When two target nodes have a similarity association, an edge is established between them. Self-loops are also introduced into the graph structure to enhance the retention of node self-information. This view is used to characterize structural or functional similarity among targets, allowing NAFF-DTI to propagate neighborhood information at the target level and alleviate the influence of sparse interaction data.

At the drug level, the drug similarity view is constructed based on the drug similarity matrix $S_{d}\in R^{N_{d}\times N_{d}}$, denoted as $G^{\left(d\right)}=(D,E^{\left(d\right)})$. If a similarity relationship exists between two drug nodes, an edge is built between them. Meanwhile, node self-connections are retained in the graph structure, so that node representations can preserve both self-features and neighborhood information during propagation. This view reflects potential associations among drugs from the perspectives of chemical structure or functional properties, and provides complementary information for drug representation learning.

For the known interaction relationships between drugs and targets, the drug–target bipartite graph view is constructed based on the interaction matrix $Y_{train}$ in the training set, denoted as $G^{\left(dt\right)}=(D\cup T,E^{\left(dt\right)})$. When $Y_{ij}=1$, an edge is established between drug node $d_{i}$ and target node $t_{j}$. This view directly reflects the observed interaction relationships and serves as the core structural basis for NAFF-DTI to learn potential association patterns.

During graph construction, the original data are filtered for consistency. Only nodes that appear in the training set and have neighborhood relationships in the similarity networks are retained, so as to avoid interference from isolated nodes during representation learning. Meanwhile, the interaction graph structure is constructed based on the training data. For the validation and test sets, negative examples are sampled from unobserved drug–target pairs to form positive and negative sample pairs for model selection and performance evaluation, whereas negative examples in the training stage are dynamically sampled during model optimization.

The resulting multi-view graph structure consists of the target similarity view, the drug similarity view, and the drug–target interaction view. These views characterize association patterns among nodes from different perspectives, providing a unified data basis for subsequent graph representation learning and node-level adaptive feature fusion.

### 2.3. Graph Encoding and Multi-View Representation Learning

After constructing the multi-view graph, NAFF-DTI performs graph representation learning on each view to obtain node embeddings in different relational spaces. Since the target similarity view, drug similarity view, and drug–target interaction view describe different association patterns, a single view is insufficient to fully represent node characteristics. Therefore, multi-view representations need to be jointly modeled and integrated. Specifically, graph convolutional encoding is first performed on each view to learn view-specific node embeddings. Then, cross-view interaction and representation aggregation are introduced to integrate homogeneous similarity information and heterogeneous interaction information. Through this process, NAFF-DTI preserves the structural characteristics of each view while exploiting complementary cross-view associations, thereby providing unified node representations for subsequent adaptive feature fusion.

#### 2.3.1. Multi-View Graph Convolutional Encoding

Before graph convolutional encoding, we first apply a lightweight self-gating operation to the initial node embeddings. This operation recalibrates feature dimensions and highlights information related to relational modeling, so that the gated node embeddings can serve as the initial inputs for subsequent graph convolutional propagation.

Based on the multi-view graph structure, graph convolutional encoding is performed on the target similarity view, the drug similarity view, and the drug–target interaction view, respectively, to learn node representations in different relational spaces. Let the graph structure corresponding to the v-th view be denoted as $G^{\left(v\right)}=(V^{\left(v\right)},E^{\left(v\right)})$, with its adjacency matrix denoted as $A^{\left(v\right)}$, and the initial node representation denoted as $H^{\left(0\right)}$. In each graph convolutional layer, node representations are updated through neighborhood information aggregation. The graph convolutional propagation used in NAFF-DTI is formulated as follows:

(1) $$H^{\left(l+1\right)}=\hat{A}H^{\left(l\right)},\;\hat{A}=D^{-\frac{1}{2}}AD^{-\frac{1}{2}}$$

where A is the adjacency matrix of the corresponding view, D is its degree matrix, and $\hat{A}$ denotes the symmetrically normalized adjacency matrix. This propagation form does not introduce additional nonlinear transformations, but preserves collaborative information in the graph structure through neighborhood aggregation [16].

The above propagation is performed separately on different views. In the target similarity view and drug similarity view, information is propagated among homogeneous nodes to characterize local structural similarity. In the drug–target interaction view, information is propagated between heterogeneous nodes to encode observed interaction patterns. Through multi-layer propagation, NAFF-DTI obtains node representations at different levels and in different relational spaces, which are further integrated in the subsequent representation aggregation and cross-view interaction process.

#### 2.3.2. Representation Aggregation and Cross-View Interaction

After multi-view graph convolutional encoding, each node obtains embedding representations from different views and different propagation layers. Since different views focus on different relational patterns, and node features gradually incorporate neighborhood information during multi-layer propagation, these representations need to be further integrated to obtain stable node representations.

During each propagation layer, the node representations in the interaction view are split according to node type and then interact with the corresponding homogeneous-view representations. For target nodes and drug nodes, the cross-view fusion process at the l-th layer can be formulated as:

(2) $$H_{t}^{\left(l\right)}=\frac{1}{2}\left(H_{t,s}^{\left(l\right)}+H_{t,ui}^{\left(l\right)}\right),H_{d}^{\left(l\right)}=\frac{1}{2}\left(H_{d,s}^{\left(l\right)}+H_{d,ui}^{\left(l\right)}\right)$$

where $H_{t,s}^{\left(l\right)}$ and $H_{d,s}^{\left(l\right)}$ denote the node representations obtained from the target similarity view and the drug similarity view at the l-th layer, respectively. $H_{t,ui}^{\left(l\right)}$ and $H_{d,ui}^{\left(l\right)}$ denote the target node representations and drug node representations split from the interaction view, respectively. Through this process, information from the similarity relationships and interaction relationships of the same node can be fused during layer-wise propagation.

To comprehensively utilize the initial representations and the multi-layer propagation results, we adopt simple averaging to aggregate node representations from different layers, thereby obtaining stable node representations. For any node, its multi-layer aggregated representation can be formulated as:

(3) $$H=\frac{1}{L+1}\sum_{l=0}^{L}H^{\left(l\right)}$$

where L denotes the number of graph convolutional layers, $H^{\left(0\right)}$ is the initial node representation, and $H^{\left(l\right)}$ is the node representation after the l-th propagation layer. This aggregation strategy can preserve both shallow neighborhood structural information and higher-order neighborhood information, avoiding information loss caused by relying only on the final-layer representation.

Through representation aggregation and cross-view interaction, NAFF-DTI obtains basic node representations from the similarity views and the interaction view. These representations are then used for subsequent node-level adaptive feature modeling and fusion.

### 2.4. Node-Level Adaptive Feature Modeling and Fusion

Based on multi-view representation learning, nodes have obtained embeddings that integrate different relational information. However, the contribution of each view varies across nodes, especially under data sparsity or long-tail distributions. Directly treating multi-view representations in a unified manner may introduce redundant information or weaken key structural features. To address this issue, we introduce a node-level adaptive feature modeling and fusion mechanism. This module takes the multi-view node representations as inputs, models cross-view representation discrepancies, and incorporates node structural properties to learn adaptive fusion weights. Specifically, discrepancy modeling and feature reweighting are first used to enhance useful auxiliary information, and node-level adaptive fusion is then performed to generate more stable and discriminative node representations.

#### 2.4.1. Cross-View Feature Discrepancy Modeling

Based on multi-view representation learning, different views often characterize the same node in different ways. The similarity views focus on structural associations among homogeneous nodes, whereas the interaction view directly reflects interaction relationships between heterogeneous nodes. Therefore, the embeddings of the same node in different views may exhibit certain semantic shifts. Relying on a single view or simple fusion is insufficient to fully capture such discrepancies.

To characterize the differences between node representations in different relational spaces, inspired by the discrepancy feature modeling and feature adaptation ideas in existing graph representation adaptation methods [17], we explicitly model cross-view features. Based on the multi-view node representations obtained in Section 2.3.2, the representations are further separated and characterized at the node level. For any node, its representation in the interaction view is denoted as $h_{ui}$, and its auxiliary-view representation is denoted as $h_{aux}$. The representation difference between them is used to characterize the cross-view shift, and the discrepancy feature is defined as:

(4) $$h_{diff}=h_{ui}-h_{aux}$$

This discrepancy representation describes the representation shift in a node in different relational spaces, and reflects the inconsistency between similarity information and interaction information. On this basis, task-related feature reweighting is introduced to adjust the importance of different feature dimensions, thereby strengthening informative features and suppressing irrelevant or noisy information.

Subsequently, the discrepancy feature is concatenated with the reweighted auxiliary-view representation and projected through a linear mapping to obtain the adapted incremental representation. The incremental representation is further combined with the original auxiliary representation to form the enhanced auxiliary representation. For simplicity, the enhanced auxiliary representation is still denoted as $h_{aux}$ in the subsequent node-level fusion process. This process provides a structured expression of cross-view discrepancy information and more discriminative input features for adaptive fusion.

#### 2.4.2. Node-Level Adaptive Fusion Mechanism

After cross-view feature discrepancy modeling, each node obtains an enhanced auxiliary representation that integrates the original representation with discrepancy information. However, the contributions of different views still vary across nodes. Therefore, an adaptive mechanism is introduced at the node level to dynamically fuse the interaction-view representation and the enhanced auxiliary representation.

The node-level adaptive weight α is automatically learned by the model according to node features. Specifically, the representation of a node in the interaction view, the enhanced auxiliary representation, the representation difference between them, and the structural degree information of the node in different views are jointly used as inputs. The fusion weight is calculated through feature concatenation and nonlinear mapping, as follows:

(5) $$\alpha =\sigma \left(MLP\left(\left[h_{ui},h_{aux},h_{ui}-h_{aux},d_{ui},d_{aux}\right]\right)\right)$$

where $h_{ui}$ denotes the node representation in the interaction view, $h_{aux}$ denotes the auxiliary representation enhanced by discrepancy modeling, and $d_{ui}$ and $d_{aux}$ denote the structural degree information of the node in the interaction view and the auxiliary view, respectively. In the implementation, the two structural degree scalars are transformed by log(1+x) and max-normalization before being fed into the gating network, so as to reduce the numerical bias caused by high-degree nodes. If the node embedding dimension is d, the input dimension of the MLP in Equation (5) is 3d+2.

The MLP in Equation (5) is implemented as a lightweight two-layer gating network. The first layer maps the 3d+2-dimensional input to a d-dimensional hidden space and uses LeakyReLU as the activation function; the second layer outputs a one-dimensional node-level fusion weight, which is further restricted to the range of (0,1) by the Sigmoid function. It should be noted that the fusion weight in this study is a node-level scalar weight rather than a feature-wise weight, and therefore controls the overall contribution ratio between the interaction-view representation and the auxiliary-view representation at the node level. No additional dropout, Batch normalization, or Layer normalization is used in this gating network. The linear-layer parameters are initialized using the default initialization strategy of the deep learning framework and are optimized end-to-end together with the other model parameters.

The final node representation is obtained by weighted fusion of information from different views:

(6) $$h=\alpha \cdot h_{ui}+(1-\alpha )\cdot h_{aux}$$

This mechanism adaptively adjusts the contribution of each view according to node representation differences and structural characteristics. When the interaction view provides more reliable structural information, a higher weight is assigned to $h_{ui}$. When the auxiliary view is more informative, the role of $h_{aux}$ is strengthened. In this way, NAFF-DTI can flexibly integrate multi-view information and obtain more stable and discriminative node representations for DTI prediction.

In terms of computational complexity, the node-level adaptive fusion module only performs feature concatenation, two linear mappings, and weighted fusion at the node-representation level, without introducing an additional graph propagation process. For each node, the additional computational cost mainly comes from the linear mapping from the 3d+2-dimensional input to the d-dimensional hidden space, with a complexity of approximately $O(d^{2})$. For all drug and target nodes, the additional complexity is approximately $O((|D|+|T|)d^{2})$. Therefore, this module mainly introduces a lightweight node-representation transformation cost and does not change the overall computational process of multi-view graph propagation.

### 2.5. Training and Optimization of NAFF-DTI

After obtaining the final representations of drug nodes and target nodes, we calculate the association score of each drug–target pair through representation matching. For drug node $d_{i}$ and target node $t_{j}$, their final representations $h_{d_{i}}$ and $h_{t_{j}}$ are used, respectively, and the prediction score is calculated by vector inner product:

(7) $${\hat{y}}_{ij}=h_{d_{i}}^{⊤}h_{t_{j}}$$

where ${\hat{y}}_{ij}$ denotes the predicted confidence score of the drug–target pair $\left(d_{i},t_{j}\right)$. This score measures the matching degree between the two types of nodes in the unified representation space. A higher score indicates a greater possibility that the two nodes have a potential interaction.

During training, NAFF-DTI is optimized based on a pairwise ranking strategy [18]. For each known interaction pair $\left(d_{i},t_{j}\right)$, a negative drug $d_{k}$ is sampled from drugs that have no observed interaction with target $t_{j}$ in the training set, and the model is expected to assign a higher score to the positive pair $\left(d_{i},t_{j}\right)$ than to the negative pair $\left(d_{k},t_{j}\right)$. Based on this, the ranking loss is defined as:

(8) $$L_{bpr}=-\sum_{\left(d_{i},t_{j},d_{k}\right)}log\;\sigma ({\hat{y}}_{ij}-{\hat{y}}_{kj})$$

where ${\hat{y}}_{ij}$ and ${\hat{y}}_{kj}$ denote the prediction scores of the positive and negative sample pairs, respectively, and σ(⋅) is the Sigmoid function. This loss enables NAFF-DTI to focus more on the relative ranking relationship between positive and negative samples during training.

To further enhance the consistency of drug representations, we introduce a drug-side contrastive learning constraint during training, aligning the drug representations from the interaction view with the fused drug representations. For the drug node set I involved in the current training batch, the contrastive learning loss is formulated as:

(9) $$L_{ssl}=-\frac{1}{|I|}\sum_{i\in I}w_{i}\;log\frac{exp(sim(z_{i}^{\left(1\right)},z_{i}^{\left(2\right)})/\tau )}{\sum_{j\in I}exp(sim(z_{i}^{\left(1\right)},z_{j}^{\left(2\right)})/\tau )}$$

where $z_{i}^{\left(1\right)}$ and $z_{i}^{\left(2\right)}$ denote the embeddings of the same drug in different representation spaces, sim(⋅) denotes the similarity calculation, τ is the temperature coefficient, and $w_{i}$ is the weight derived from the popularity of the drug node. This term corresponds to the alignment constraint for drug nodes across different representation spaces. Since the drug side contains richer structural similarity information, whereas the target-side similarity information is relatively sparse, we only introduce the contrastive learning constraint on drug nodes to provide an auxiliary constraint for drug representation alignment.

Meanwhile, to prevent node representations from becoming excessively large and to improve training stability, an embedding regularization term is introduced. An L2 constraint is imposed on the representations of target nodes, positive drug nodes, and negative drug nodes involved in each training batch, which can be formulated as:

(10) $$L_{reg}=\sum ||\theta |{|}^{2}$$

where θ denotes the node embedding representations involved in the current training batch.

In addition, for the node-level adaptive fusion module, we introduce a lightweight gate regularization term to constrain the distribution of adaptive fusion weights and prevent them from rapidly or excessively leaning toward a single view during training. This regularization term penalizes the deviation between the fusion weight and the middle value, allowing the model to maintain a certain degree of fusion flexibility during learning. It is formulated as:

(11) $$L_{gate}=\frac{1}{|V|}\sum_{i\in V}{\left(0.5-\alpha_{i}\right)}^{2}$$

where $\alpha_{i}$ denotes the adaptive fusion weight of node i. This regularization term does not fix the fusion weight, but constrains overly polarized weight distributions during optimization.

The final optimization objective is defined as:

(12) $$L=\frac{1}{B}\left(L_{bpr}+\lambda_{r}L_{reg}\right)+\lambda_{ssl}L_{ssl}+\lambda_{m}L_{gate}$$

where B is the batch size, and $\lambda_{r}$, $\lambda_{ssl}$, and $\lambda_{m}$ control the weights of the regularization term, contrastive learning loss, and gate regularization term, respectively. The overall optimization objective consists of the BPR loss, embedding regularization term, drug-side contrastive learning loss, and adaptive gate regularization term.

The parameters of NAFF-DTI are updated using the Adam optimizer [19]. During training, model selection is performed according to the AUC and AUPR on the validation set. If the validation performance does not show an obvious improvement for several consecutive epochs, training is stopped early, and the model with the best validation performance is loaded for test set evaluation. In the testing stage, the learned target and drug representations are directly used to calculate prediction scores, and AUC and AUPR are further computed as performance metrics.

### 2.6. Experimental Settings

#### 2.6.1. Datasets

To comprehensively evaluate the performance of the proposed model, we first construct a multi-source heterogeneous information fusion dataset to describe interaction relationships between drugs and protein targets [20]. To avoid ambiguity with the concept of drug–target interaction (DTI), this dataset is denoted as the IDTI (Integrated Drug–Target Interaction) dataset in this paper. In addition, commonly used benchmark datasets in the DTI prediction field are selected for comparative experiments, including the Luo dataset [21] and three subsets proposed by Yamanishi, namely GPCR (G protein-coupled receptors), Enzyme, and IC (ion channels) [22]. It should be noted that the nuclear receptors subset in the Yamanishi dataset is not used in this paper because of its relatively small sample size and limited application. The descriptive statistics of each dataset are shown in Table 1. The differences in dataset scale help evaluate model performance from different data-size perspectives. These datasets have been widely used in related studies and have good representativeness and comparability. In terms of data processing, we follow the preprocessing procedure used in previous work, including sample filtering, data splitting, and negative sample construction, to ensure the comparability and fairness of experimental results [20].

#### 2.6.2. Baseline Methods

We selected NeoDTI [23], GENNIUS [24], DDGAE [25], NASNet-DTI [26], FGS_GRMF_ [27], MKDTI [28], and MGACL [20] as baseline methods and compared them with the proposed NAFF-DTI. These baselines cover several representative modeling strategies in DTI prediction, including heterogeneous network- and graph neural network-based representation learning, similarity fusion- and multiple kernel learning-based prediction, and graph contrastive learning-based representation enhancement. Therefore, they provide complementary perspectives for evaluating the predictive performance of NAFF-DTI.

Specifically, NeoDTI learns drug and target representations through neighborhood information aggregation and topology-preserving learning in heterogeneous networks. GENNIUS generates drug and protein node embeddings using graph neural networks and predicts potential interactions with a neural network classifier. DDGAE enhances heterogeneous network representation using dynamically weighted residual graph convolution and a graph convolutional autoencoder. NASNet-DTI adjusts the graph information propagation depth according to local node structures to alleviate the over-smoothing problem caused by fixed propagation layers. FGS_GRMF_ integrates multi-source similarity information through fine-grained selective similarity fusion and performs DTI prediction with GRMF, whereas MKDTI extracts multi-layer node embeddings using a graph attention network and performs prediction through multiple kernel fusion and a dual Laplacian regularized least squares framework. MGACL enhances drug and target representations through meta-graph association-aware contrastive learning and personalized knowledge transfer.

#### 2.6.3. Evaluation Metrics and Implementation Details

We adopt AUC (Area Under the ROC Curve) and AUPR (Area Under the Precision–Recall Curve) as evaluation metrics. AUC reflects the overall ranking ability of the model, while AUPR is more informative under sparse and imbalanced biological network settings where non-interaction pairs greatly outnumber interaction pairs [29].

In the experimental setting, we evaluate the model using 5-fold cross-validation. In each fold, the data are divided into a training set and a test set, with the test set accounting for 20%. A further 10% of samples from the training set are used as the validation set for model selection, and the final performance is reported on the test set. To ensure evaluation stability, the final performance is reported as the mean and standard deviation over the five folds.

To ensure comparability among different methods, we did not directly cite the experimental results reported in the original baseline papers. Instead, all baseline methods were rerun or adapted under the same datasets, five-fold splits, and evaluation protocol. In each fold, only the known interactions in the training set were used to construct the training interaction matrix or training graph, the validation set was used for model selection, and the test set was used only for final performance evaluation. For all methods, AUC and AUPR were calculated on the same test positive and test negative samples, and the final results were reported as the mean and standard deviation over the five folds.

In terms of implementation, NAFF-DTI is implemented using the PyTorch framework (version 2.7.1+cu128), and the Adam optimizer is used for parameter updating. Model parameters are tuned within candidate ranges, and the final settings are determined according to validation performance. The node embedding dimension d is set to 128, the learning rate is set to 0.0001, the batch size is set to 16, the number of graph neural network layers is set to 3, the maximum number of training epochs is set to 400, and the early stopping patience is set to 5. The gate regularization weight in the node-level adaptive fusion module is set to 0.001, the cross-view feature adaptation weight is set to 1.0, and the weight of the contrastive learning loss in the overall objective function is set to 0.3. During training, the model is evaluated on the validation set after each epoch. When the validation AUC or AUPR improves, the current model is saved. If no obvious improvement is achieved for 5 consecutive epochs, training is stopped early, and the model with the best validation performance is loaded for testing. To improve training stability, gradient clipping is used during model training, with the maximum gradient norm set to 20.

In terms of data splitting and sample construction, negative samples are dynamically constructed during training based on the BPR strategy. In the testing stage, one negative sample is randomly sampled for each positive sample to construct a 1:1 positive–negative sample set. Meanwhile, the test set is filtered to ensure that both drugs and proteins in the test samples appear in the training set, thereby ensuring the validity of the evaluation results.

## 3. Results

### 3.1. Overall Performance Comparison

The AUC and AUPR results of different methods on different datasets are shown in Table 2 and Table 3. Overall, NAFF-DTI achieves the best AUC and AUPR on all five datasets, indicating that the method has relatively stable predictive performance across datasets of different scales and types.

From the perspective of method mechanisms, the performance differences mainly arise from how different methods model multi-source information. NeoDTI, GENNIUS, DDGAE, and NASNet-DTI mainly rely on heterogeneous networks or graph neural networks for representation learning, and can extract drug and target features through neighborhood aggregation, graph encoding, or node propagation mechanisms. Therefore, these methods generally perform well on datasets such as Enzyme and IC, where the relational structures are relatively easier to learn. NASNet-DTI further adjusts the propagation depth according to local node structures, and also achieves good results on the GPCR, Enzyme, and IC datasets. However, these methods are still mainly based on graph propagation or node embedding learning, and insufficiently consider the contribution differences in different information sources across different nodes. Therefore, on datasets such as IDTI and Luo, where data scale, sparsity, or relational structure changes, their performance advantages are not fully stable.

FGS_GRMF_ and MKDTI mainly utilize drug similarity, target similarity, and the interaction matrix from the perspective of similarity fusion or multiple kernel learning. Similarity information can provide complementary information for sparse DTI data. Therefore, these methods show certain effectiveness on datasets such as GPCR, Enzyme, and IC, and MKDTI also achieves a relatively high AUPR on the Luo dataset. However, on the IDTI dataset, the AUC values of FGS_GRMF_ and MKDTI are relatively low. This indicates that when the node scale is larger and the relational structure is more complex, relying only on similarity integration or kernel-matrix-level fusion is insufficient to fully characterize complex heterogeneous information, and it is also difficult to dynamically adjust the importance of different information sources according to node status.

In contrast, NAFF-DTI transforms multi-source information fusion into a node-level adaptive selection problem. By introducing cross-view discrepancy modeling and a node-level adaptive fusion mechanism, NAFF-DTI allows different views to make different contributions for different nodes, thereby strengthening useful information and reducing the influence of redundant or noisy views. When interaction information is relatively sufficient, NAFF-DTI can make greater use of association patterns in the interaction structure. When interaction information is insufficient, the drug similarity and target similarity views can provide complementary information. This adaptive fusion strategy improves the discriminative ability of node representations, enabling NAFF-DTI to maintain stable performance across datasets with different relational complexities and data scales.

Compared with MGACL, NAFF-DTI places more emphasis on node-level adaptive feature fusion across different views, rather than mainly relying on contrastive learning to enhance representation consistency. MGACL performs well on the IDTI and Enzyme datasets and also shows certain competitiveness on the Luo and IC datasets, but its AUC and AUPR on the GPCR dataset are clearly lower than those of most other methods. The GPCR dataset is relatively small and contains a limited number of known interactions. Therefore, the sample relationships and structural contexts required by contrastive learning may be insufficient, which limits its representation enhancement effect. NAFF-DTI directly adjusts the contribution of different views through a node-level feature selection mechanism, reducing its dependence on contrastive sample relationships. Therefore, it can still maintain high performance on small-scale and sparse datasets such as GPCR.

From the perspective of datasets, the advantage of NAFF-DTI on the IDTI dataset shows that it can effectively model complex heterogeneous information. Its stable performance on the Luo dataset indicates that the method has good applicability on typical benchmark data. On the Yamanishi datasets, especially the GPCR subset, the performance improvement suggests that NAFF-DTI can still maintain a certain degree of stability under small-sample conditions.

Overall, baseline methods usually rely on one major modeling mechanism. Therefore, they can achieve good results on datasets suitable for that mechanism, but their performance may fluctuate when the data scale, sparsity, or relational structure changes. By introducing an adaptive feature fusion mechanism, NAFF-DTI achieves a better balance between multi-source information utilization and noise suppression, thereby showing stable and competitive performance under different data distributions.

To supplement the overall performance comparison, one-sided paired Wilcoxon signed-rank tests were performed between NAFF-DTI and the strongest baseline for each dataset and each metric based on the five-fold results. Detailed results are provided in Supplementary Table S1.

In addition, considering that the balanced 1:1 negative sampling setting may lead to optimistic performance estimates, especially for AUPR, we conducted supplementary evaluations under 1:10 and all-candidate negative settings on the IDTI and Luo datasets to assess the ranking ability of NAFF-DTI in more imbalanced and realistic candidate spaces, with detailed results provided in Supplementary Table S2.

### 3.2. Ablation Study

Based on the overall performance comparison, we further conduct ablation experiments to analyze the contribution of different components in NAFF-DTI. According to the model structure described in Section 2, three ablation variants are constructed around multi-view information modeling, cross-view discrepancy modeling, and feature enhancement.

Specifically, based on the complete NAFF-DTI model, auxiliary-view-related information is first removed, and only the interaction view is retained for representation learning (*w*/*o*-aux), which is used to evaluate the role of multi-view information modeling. Second, while retaining the multi-view structure, the cross-view discrepancy modeling and feature adaptation process is removed, and adaptive fusion is performed only based on the original multi-view representations (*w*/*o*-adapt), which is used to analyze the contribution of cross-view discrepancy modeling and feature adaptation. Finally, under the complete fusion framework, the residual fusion in the enhanced auxiliary representation is removed, and only the adapted representation is retained for subsequent fusion (*w*/*o*-enhance), which is used to evaluate the influence of the feature enhancement strategy.

Under the same experimental settings, the above model variants are compared with the complete model to systematically analyze the roles of multi-view information utilization, adaptive fusion, and feature enhancement in NAFF-DTI. Considering that the IDTI and Luo datasets represent a complex multi-source relational scenario and a medium-scale typical scenario, respectively, we conduct ablation experiments on these two datasets. The experimental results are shown in Table 4.

As shown in Table 4, different ablation variants show varying degrees of decrease in average performance on both datasets, with relatively small standard deviations, indicating that the core architectural components evaluated in Table 4 contribute consistently to the final performance. Among them, the model without auxiliary-view information (*w*/*o*-aux) shows the most significant performance decline, with both AUC and AUPR clearly decreasing on the IDTI and Luo datasets. This indicates that relying only on the interaction view is insufficient to fully characterize the complex relationships between drugs and targets, and that multi-view information modeling plays a key role in alleviating data sparsity and enriching structural information.

When the multi-view structure is retained, removing the cross-view discrepancy modeling and feature adaptation process (*w*/*o*-adapt) also leads to performance degradation, although the decrease is relatively smaller. This indicates that differences indeed exist among different views, and that cross-view discrepancy modeling provides useful discriminative information for adaptive fusion. By assigning weights according to cross-view representations and structural features, the node-level adaptive fusion mechanism improves the flexibility of multi-source information utilization.

For the cross-view feature enhancement module (*w*/*o*-enhance), the performance decline is relatively limited, because cross-view discrepancy modeling and node-level adaptive fusion are still retained. This indicates that the enhancement strategy mainly helps preserve useful auxiliary structural information, while the main fusion framework remains effective.

Overall, the ablation results confirm that multi-view information modeling provides the basic structural support, cross-view discrepancy modeling and adaptive fusion improve information utilization, and the feature enhancement strategy helps preserve useful structural and discrepancy information.

In addition to the main ablation study, source-level ablation experiments were conducted by separately removing the target similarity view and the drug similarity view, with detailed results provided in Supplementary Table S3.

### 3.3. Hyperparameter Sensitivity Analysis

**Figure 2:**
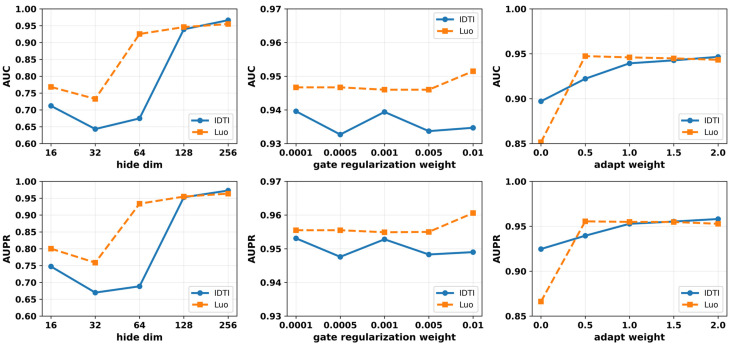
Sensitivity analysis of key hyperparameters on IDTI and Luo datasets.

After validating the effectiveness of the model structure, we further analyze the influence of key hyperparameters on NAFF-DTI. According to the model design in Section 2, three parameters are considered: the embedding dimension, the gate regularization strength in node-level adaptive fusion, and the feature adaptation weight in cross-view discrepancy modeling. By varying these parameters while keeping the other settings unchanged, we evaluate the stability of NAFF-DTI on the IDTI and Luo datasets. The results are shown in Figure 2.

As shown in Figure 2, different hyperparameters have different effects on model performance. For the embedding dimension, AUC and AUPR generally improve as the dimension increases from 16 to 128, indicating that low-dimensional representations are insufficient to capture complex multi-view relationships. When the dimension further increases to 256, the performance gain becomes limited. Considering both performance and computational cost, the embedding dimension is set to 128.

For the gate regularization strength, model performance remains relatively stable under different values, indicating that this term mainly constrains the distribution of fusion weights and prevents excessive dependence on a single view, rather than dominating performance changes. Since 0.001 shows stable performance on both datasets, it is adopted as the default setting.

For the feature adaptation weight in cross-view discrepancy modeling, its influence is more obvious. When the weight is 0, performance decreases clearly, suggesting that discrepancy information is important for representation learning. As the weight increases, model performance improves and remains at a relatively high level within a certain range. However, an excessively large weight does not further improve performance and may affect the balance of information fusion. Therefore, 1.0 is selected as the feature adaptation weight.

Overall, NAFF-DTI maintains stable performance within a reasonable parameter range, showing a certain degree of parameter robustness.

### 3.4. Generalization Ability Analysis Based on Repeated Holdout Validation

After the overall performance comparison and ablation analysis, we further evaluate the stability and generalization ability of NAFF-DTI under different data splitting conditions using repeated holdout validation. Unlike fixed splitting or cross-validation, repeated holdout validation randomly divides the dataset multiple times and examines model performance under different training–test splits.

**Figure 3:**
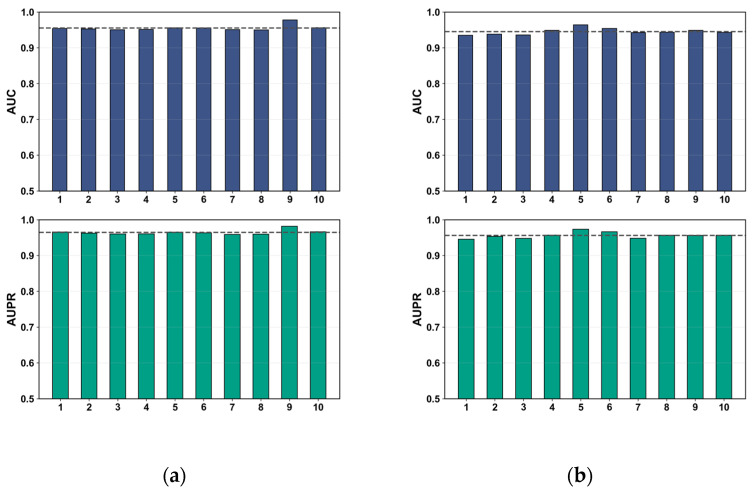
Generalization performance of NAFF-DTI under repeated holdout validation on (**a**) IDTI dataset and (**b**) Luo dataset. Each bar represents one independent random split, and the dashed line indicates the mean performance over 10 runs. On the IDTI dataset, the test AUC and AUPR were 0.9553 ± 0.0078 and 0.9643 ± 0.0063, respectively. On the Luo dataset, the test AUC and AUPR were 0.9452 ± 0.0084 and 0.9560 ± 0.0081, respectively.

Specifically, in each experiment, the dataset is randomly divided into a training set and a test set, with the test set accounting for 10%. Then, 10% of the samples from the training set are used as the validation set for model selection and early stopping. Multiple random seeds are used to repeat the splitting process, and the model is independently trained under each split. All repeated experiments adopt the same parameter settings and optimization strategy. The best model is selected according to validation performance and evaluated on the corresponding test set. Finally, the mean and standard deviation of multiple experimental results are calculated. Considering that the IDTI and Luo datasets represent a complex multi-source relational scenario and a medium-scale typical scenario, respectively, repeated holdout validation is conducted on these two datasets. The results are shown in Figure 3.

As shown in Figure 3, NAFF-DTI maintains stable performance on both datasets under different random splits. On the IDTI dataset (Figure 3a), AUC and AUPR remain at high levels across 10 random splits, with relatively small fluctuations. This indicates that NAFF-DTI can learn stable representations under complex multi-source relationships and uneven data distributions. On the Luo dataset (Figure 3b), the results are also concentrated around the mean values, suggesting that NAFF-DTI can maintain consistent performance on medium-scale benchmark data. Overall, repeated holdout validation shows that NAFF-DTI is not sensitive to data splitting and has good generalization ability under different data distributions.

### 3.5. Performance Analysis Under Different Evaluation Scenarios

Since unknown nodes and long-tail distributions commonly exist in practical DTI prediction tasks, overall performance based only on random splitting is insufficient to comprehensively evaluate model capability. Therefore, we analyze the performance of NAFF-DTI from different evaluation scenarios, including cold-start and long-tail scenarios, to characterize the behavior of the model under incomplete information and uneven data distributions.

#### 3.5.1. Cold-Start Scenario Analysis

In practical DTI prediction tasks, models need to deal with new drugs or new targets that have not appeared during training. Therefore, evaluation results based only on random splitting cannot fully reflect their generalization ability [30]. To this end, we construct cold-start evaluation scenarios on the Luo dataset to analyze the prediction performance under unknown-node conditions.

Specifically, referring to the evaluation setting under unseen drugs, unseen targets, and both unseen conditions [31], we implement cold-start settings by constraining data splitting. Nodes with sufficient interaction information are first selected as the candidate set. Then, a subset of drugs or proteins is randomly selected as test nodes, and their related interactions are removed from the training data. For one-sided cold-start settings, only one side of the nodes is retained in the training set, forming the Drug Cold-start and Protein Cold-start settings, respectively. Furthermore, nodes on both sides are removed simultaneously to construct the Both Cold-start setting, which simulates a stricter unknown-node prediction condition.

**Figure 4:**
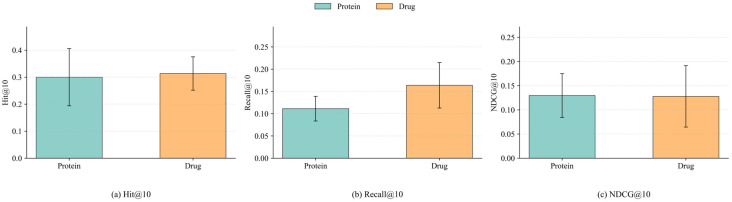
Performance under different cold-start settings on the Luo dataset.

Under this setting, NAFF-DTI needs to complete prediction without historical interaction information, relying only on node features and graph structural information, which poses higher requirements for representation learning ability. Since cold-start evaluation focuses on whether true interactions are ranked near the top of the candidate list, we adopt Hit@10, Recall@10, and NDCG@10 as Top-K ranking metrics, following the common evaluation practice in cold-start recommendation tasks [32]. The results are shown in Figure 4.

Figure 4 shows the performance under the Protein Cold-start and Drug Cold-start settings. It can be observed that all metrics decrease significantly under cold-start conditions. Further comparison shows that Drug Cold-start performs better than Protein Cold-start overall, and the difference is more obvious in terms of Recall@10. This indicates that NAFF-DTI faces greater prediction difficulty when unknown nodes are located on the target side, probably because protein-side similarity information is relatively sparse in the current data, making effective target representations harder to obtain under cold-start conditions. In contrast, the richer similarity information on the drug side helps maintain a certain level of predictive performance.

It should be noted that, under the Both Cold-start setting, both endpoints of the test edges do not appear in the training set. As a result, NAFF-DTI lacks effective prior interaction information for both drugs and targets. In our experiments, the Top-K ranking metrics under this setting were all zero, with Hit@10, Recall@10, and NDCG@10 equal to 0 across repeated runs. Therefore, the Both Cold-start results are not plotted in Figure 4 to avoid an uninformative zero-valued bar, and the corresponding numerical results are provided in Supplementary Table S4. Overall, as the cold-start constraint becomes stronger, the performance of NAFF-DTI gradually decreases, further reflecting the difficulty of DTI prediction under unseen-node conditions.

#### 3.5.2. Long-Tail Scenario Analysis

In drug–target interaction networks, node interactions often show an imbalanced distribution, where a small number of nodes have many known interactions while many nodes contain limited interaction information. Similar long-tail effects have been shown to bias recommendation models toward high-frequency items and weaken their performance on low-frequency items [33]. To this end, we construct a long-tail evaluation scenario on the Luo dataset to analyze the performance of NAFF-DTI under different interaction-frequency conditions.

**Figure 5:**
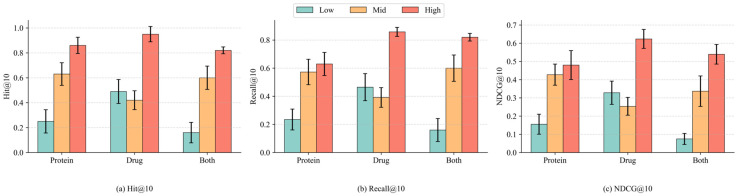
Performance across different groups under the long-tail setting on the Luo dataset.

Specifically, after regular training, test samples are grouped according to the number of interactions of nodes in the training set. Based on interaction frequency, nodes are divided into low-frequency (low), medium-frequency (mid), and high-frequency (high) groups. The Drug and Protein settings are grouped according to the interaction frequency of the corresponding nodes, while the Both setting jointly considers interaction information from both drug and target sides. All experiments are repeated under multiple random seeds to reduce the influence of data splitting. Hit@10, Recall@10, and NDCG@10 are used as evaluation metrics, and the results are shown in Figure 5.

Figure 5 shows the performance of different groups under the Protein, Drug, and Both settings. The results show that all metrics generally improve as node interaction frequency increases, with the high group outperforming the mid group and the mid group outperforming the low group. This indicates that NAFF-DTI can learn more stable representations for nodes with sufficient interaction information, while its performance is limited on low-frequency nodes.

Further comparison shows that group differences are more obvious in the Drug setting, especially between the low and high groups, indicating that NAFF-DTI is more sensitive to interaction-frequency changes on the drug side. In contrast, the differences in the Protein setting are relatively smaller. Under the Both setting, overall performance is lower than that in the one-sided settings, and the low group shows the most obvious decline. This suggests that when both drugs and targets are located in low-frequency regions, NAFF-DTI has difficulty obtaining effective constraints, leading to further performance degradation. Overall, the results show that lower node interaction frequency leads to higher prediction difficulty, and long-tail distribution has a clear impact on model performance.

To further examine the role of node-level adaptive fusion under long-tail conditions, we compared NAFF-DTI with its *w*/*o*-adapt variant under the same Luo long-tail settings. Supplementary Table S5 reports the corresponding Top-K results for the low-frequency groups. NAFF-DTI outperformed *w*/*o*-adapt in the low-frequency groups under the protein-, drug-, and both-side long-tail settings, indicating that node-level adaptive fusion improves prediction for low-frequency nodes and partially mitigates long-tail bias. However, the low-frequency groups remained performance-limited, suggesting that the long-tail problem was not fully eliminated.

### 3.6. Case Study

In addition to the above quantitative experiments, this study further selected representative drug–target pairs from the prediction results for case analysis, aiming to examine whether NAFF-DTI can prioritize plausible candidate associations from the unannotated interaction space. In DTI prediction tasks, drug–target pairs not recorded as positive samples do not necessarily indicate true non-interactions, but usually mean that relevant evidence has not yet been included in the current dataset. Therefore, if high-ranked candidates can be supported by external annotations or complementary evidence, it may indicate that the model has a certain ability to prioritize biologically plausible DTI candidates. However, such evidence should be interpreted as support for computational hypothesis generation rather than as direct biological validation.

Specifically, this study first ranked the unannotated drug–target pairs according to the prediction scores generated by NAFF-DTI, thereby obtaining high-confidence candidate results. Furthermore, DrugBank 6.0 [34] was queried on 8 May 2026 to examine whether the high-confidence candidate associations were supported by external database records. Ten representative results were selected to demonstrate the candidate prioritization ability of the model, as shown in Table 5. Importantly, DrugBank records were used only for post hoc evidence querying after the prediction scores had been generated. They were not used as input features, training labels, validation criteria, or ranking criteria in NAFF-DTI. The candidate ranking was fully determined by the learned model scores, and DrugBank was consulted only after ranking to provide external annotation-level support.

The results in Table 5 show that NAFF-DTI can prioritize candidate drug–target associations that are supported by external database records but are not annotated as positive samples in the current dataset. Among them, several candidate associations have corresponding records in DrugBank, suggesting that some high-ranked predictions are consistent with external database annotations that are absent from the current benchmark labels. Nevertheless, because biomedical databases may share overlapping sources, DrugBank evidence is regarded here as annotation-level support rather than independent experimental validation. Considering that elzovantinib–EGFR ranked highly in the prediction results and was not directly recorded as an interaction relationship in DrugBank, this drug–target pair was selected as a key case and further analyzed using molecular docking binding energy, three-dimensional structural visualization, and two-dimensional interaction diagrams.

**Figure 6:**
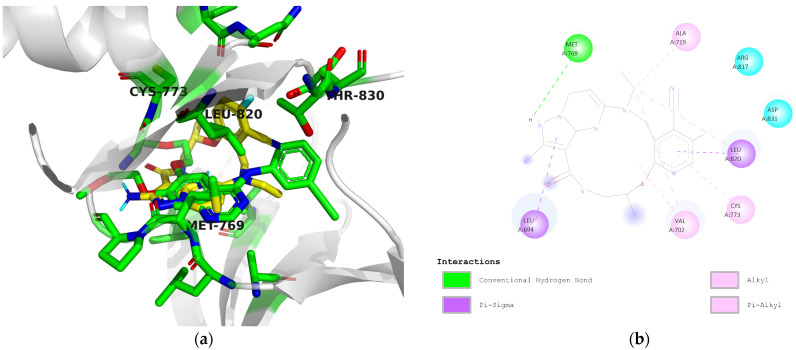
Molecular docking and interaction visualization of elzovantinib with EGFR. (**a**) Three-dimensional binding conformation; (**b**) two-dimensional interaction diagram.

To further analyze the predicted elzovantinib–EGFR relationship, the ligand information of elzovantinib was obtained from PubChem [35] , and the EGFR target information was checked using UniProt [36]. The EGFR receptor structure used for docking was obtained from the Protein Data Bank (PDB ID: 1M17, chain A), corresponding to the human EGFR kinase domain annotated in UniProt entry P00533. After standard receptor and ligand preparation, molecular docking was performed using AutoDock Vina 1.2.7 [37,38]. The docking grid was centered at $\left(x,y,z)=(21.857,\;0.260,\;52.761\right)$, corresponding to the selected local binding region of EGFR. The grid box size was set to 22×22×22 Å, the exhaustiveness was set to 8, and 9 binding modes were generated within an energy range of 3 kcal/mol. The best predicted docking pose showed a binding energy of −8.975 kcal/mol, suggesting a locally plausible docking conformation for this candidate pair. After obtaining the optimal binding conformation, the docking result was further analyzed using three-dimensional structural visualization and two-dimensional interaction diagrams, following a commonly used docking visualization workflow [39], and the corresponding results are shown in Figure 6. From the two-dimensional interaction diagram, it can be seen that elzovantinib forms a conventional hydrogen bond with surrounding residues in the binding pocket, while π-σ, alkyl, and π-alkyl hydrophobic interactions are also present. These interactions help stabilize the conformation of the ligand at the binding site. The three-dimensional visualization results show that elzovantinib can fit into the local binding region of EGFR and maintain a close spatial distance with surrounding amino acid residues.

Although DrugBank does not directly record the elzovantinib–EGFR interaction, previous studies have shown that elzovantinib is a multi-target tyrosine kinase inhibitor targeting MET, SRC, and CSF1R [40]. Meanwhile, SRC can regulate EGFR activation and amplify its downstream signaling, and is also involved in EGFR-related signal transduction processes [41]. Therefore, the relationship between elzovantinib and EGFR may not be completely isolated, but may have an indirect association through SRC-related signaling regulatory networks. Taken together, the database annotations, docking result, and literature evidence provide complementary support for the biological plausibility of the elzovantinib–EGFR candidate. However, these results should be interpreted as computational and annotation-level evidence rather than experimental validation. Moreover, co-crystallized ligand re-docking, RMSD-based docking protocol validation, and molecular dynamics simulations were not performed in the current study; thus, the docking result should be interpreted as preliminary structural plausibility evidence rather than rigorous validation of binding stability. This case suggests that NAFF-DTI may help prioritize plausible DTI candidates from the unannotated space for further independent biological investigation.

## 4. Discussion

The experimental results suggest that NAFF-DTI benefits from node-level adaptive fusion when modeling sparse and heterogeneous DTI data. By combining cross-view representation discrepancies with node structural information, the model can make complementary use of drug similarity, target similarity, and known interaction views. This design helps reduce the influence of uneven information contributions across different nodes and relational views.

Nevertheless, several limitations should be noted. First, the negative samples used in this study were sampled from unobserved drug–target pairs rather than experimentally confirmed non-interactions, which may introduce false-negative noise and lead to optimistic performance estimates under sparse and imbalanced DTI scenarios. Second, although NAFF-DTI shows stable performance under repeated holdout validation, the scenario-specific analyses indicate that strict cold-start and long-tail conditions remain challenging. In particular, when both endpoints of a test interaction are unseen during training, the model lacks sufficient relational evidence, and low-frequency nodes still provide limited supervision and neighborhood information. These results suggest that node-level adaptive fusion can alleviate information imbalance to some extent, but cannot fully overcome the limitations caused by insufficient relational evidence.

In addition, NAFF-DTI is primarily designed for large-scale DTI candidate prioritization based on relational similarity and interaction graphs. The current framework does not explicitly encode molecular descriptors or structure-based features, such as stereochemistry, three-dimensional conformations, electrostatic interactions, hydrogen-bond patterns, or physicochemical properties. Therefore, NAFF-DTI should be viewed as a candidate prioritization model rather than a mechanistic model for atomic-level molecular recognition.

The interpretation of database-supported case studies also requires caution. DrugBank 6.0 was used only as a post hoc annotation resource after model prediction and was not used as an input feature, training label, validation criterion, or ranking criterion. However, possible overlap among biomedical databases may still introduce circularity when benchmark datasets are directly or indirectly derived from related public database annotations. Therefore, DrugBank-supported candidates should be regarded as annotation-level support rather than independently validated interactions. Although PubChem/UniProt-based structural information, molecular docking, and literature evidence related to SRC–EGFR signaling were further used to provide complementary support for the representative elzovantinib–EGFR case, these analyses remain computational and indirect. Future work may explore more reliable strategies for modeling unlabeled drug–target pairs, such as positive–unlabeled learning or confidence-weighted negative sampling, and incorporate structure-aware molecular representations and independent biological assays to improve the reliability and interpretability of DTI candidate prioritization.

## 5. Conclusions

In this study, we proposed NAFF-DTI, a node-level adaptive feature fusion network based on multi-view graphs, for drug–target interaction prediction. NAFF-DTI uniformly models drug similarity, target similarity, and known interactions as a multi-view graph structure, and dynamically adjusts the contribution of different views to node representations through cross-view feature discrepancy modeling and node-level adaptive fusion. In this way, the model improves the utilization of multi-source relational information under sparse and heterogeneous data conditions. Experimental results show that NAFF-DTI achieves competitive predictive performance on multiple benchmark datasets and can prioritize biologically plausible candidate drug–target associations from the unannotated candidate space, suggesting that this method may provide computational support for DTI candidate screening and repurposing-oriented hypothesis generation.

## Figures and Tables

**Table 1:** Descriptive statistics of the datasets.

	IDTI Dataset	Luo Dataset	Yamanishi Dataset		
			GPCR	Enzyme	IC
Drug	1269	549	223	445	210
Protein	1565	421	95	664	204
Known Interaction	5222	1920	635	2926	1476
Sparsity	99.737%	99.170%	97.003%	99.010%	96.555%

**Table 2:** Overall performance comparison on the IDTI and Luo datasets.

Model	IDTI Dataset		Luo Dataset	
	AUC	AUPR	AUC	AUPR
NeoDTI	0.8339 ± 0.0136	0.8370 ± 0.0109	0.8018 ± 0.0302	0.8036 ± 0.0359
GENNIUS	0.8709 ± 0.0052	0.8740 ± 0.0102	0.8484 ± 0.0300	0.8571 ± 0.0280
DDGAE	0.8703 ± 0.0075	0.8895 ± 0.0069	0.8905 ± 0.0275	0.9047 ± 0.0241
NASNet-DTI	0.9018 ± 0.0036	0.9152 ± 0.0025	0.8835 ± 0.0311	0.8992 ± 0.0284
FGS_GRMF_	0.8136 ± 0.0082	0.8627 ± 0.0072	0.8452 ± 0.0129	0.8855 ± 0.0100
MKDTI	0.8153 ± 0.0120	0.8694 ± 0.0099	0.8737 ± 0.0129	0.9111 ± 0.0105
MGACL	0.8799 ± 0.0076	0.9115 ± 0.0062	0.8722 ± 0.0085	0.8999 ± 0.0074
**NAFF-DTI**	**0.9394 ± 0.0114**	**0.9528 ± 0.0094**	**0.9460 ± 0.0094**	**0.9549 ± 0.0089**

Values are reported as mean ± standard deviation over five folds. The best results are highlighted in bold.

**Table 3:** Overall performance comparison on the Yamanishi benchmark datasets.

Model	GPCR		Enzyme		IC	
	AUC	AUPR	AUC	AUPR	AUC	AUPR
NeoDTI	0.8424 ± 0.0341	0.8324 ± 0.0462	0.9286 ± 0.0095	0.9059 ± 0.0088	0.9444 ± 0.0117	0.9452 ± 0.0128
GENNIUS	0.8621 ± 0.0068	0.8445 ± 0.0132	0.9498 ± 0.0054	0.9468 ± 0.0096	0.9277 ± 0.0098	0.9227 ± 0.0152
DDGAE	0.9019 ± 0.0266	0.9033 ± 0.0382	0.9733 ± 0.0060	0.9783 ± 0.0038	0.9701 ± 0.0066	0.9706 ± 0.0088
NASNet-DTI	0.9138 ± 0.0115	0.9240 ± 0.0071	0.9677 ± 0.0056	0.9749 ± 0.0041	0.9707 ± 0.0067	0.9756 ± 0.0057
FGS_GRMF_	0.9006 ± 0.0103	0.9092 ± 0.0168	0.9270 ± 0.0060	0.9512 ± 0.0038	0.9301 ± 0.0074	0.9459 ± 0.0096
MKDTI	0.8974 ± 0.0196	0.9248 ± 0.0115	0.9404 ± 0.0059	0.9626 ± 0.0033	0.9580 ± 0.0064	0.9723 ± 0.0049
MGACL	0.6102 ± 0.0266	0.6139 ± 0.0442	0.9622 ± 0.0100	0.9726 ± 0.0072	0.8966 ± 0.0192	0.9191 ± 0.0172
**NAFF-DTI**	**0.9572 ± 0.0151**	**0.9620 ± 0.0133**	**0.9928 ± 0.0031**	**0.9941 ± 0.0025**	**0.9889 ± 0.0042**	**0.9914 ± 0.0029**

Values are reported as mean ± standard deviation over five folds. The best results are highlighted in bold.

**Table 4:** Ablation study results of NAFF-DTI on IDTI and Luo datasets.

Model	IDTI Dataset		Luo Dataset	
	AUC	AUPR	AUC	AUPR
*w*/*o*-aux	0.8549 ± 0.0094	0.8950 ± 0.0066	0.8262 ± 0.0112	0.8605 ± 0.0121
*w*/*o*-adapt	0.8974 ± 0.0115	0.9246 ± 0.0095	0.9126 ± 0.0066	0.9297 ± 0.0041
*w*/*o*-enhance	0.9384 ± 0.0150	0.9501 ± 0.0130	0.9390 ± 0.0045	0.9494 ± 0.0045
NAFF-DTI	0.9394 ± 0.0114	0.9528 ± 0.0094	0.9460 ± 0.0094	0.9549 ± 0.0089

Values are reported as mean ± standard deviation over five folds.

**Table 5:** Representative high-confidence candidate drug–target pairs prioritized by NAFF-DTI.

Rank	Drug ID	Drug Name	Target ID	Target Name
1	DB18918	elzovantinib	P08581	MET
2	DB20960	mazapertine	P14416	DRD2
3	DB17097	vorasidenib	O43837	IDH3B
4	DB00402	eszopiclone	P34903	GABRA3
5	DB18918	elzovantinib	P00533	EGFR
6	DB00540	nortriptyline	P31645	SLC6A4
7	DB09068	vortioxetine	P46098	HTR3A
8	DB02567	pd173955	P12931	SRC
9	DB14860	ensartinib	P29317	EPHA2
10	DB14011	nabiximols	Q8NER1	TRPV1

## Data Availability

The source code of NAFF-DTI and the processed IDTI dataset used in this study are publicly available at https://github.com/XieLinOUC/NAFF-DTI (accessed on 10 June 2026). The IDTI dataset was derived from the dataset files provided in previous work [20] and processed following the same preprocessing procedure. The Luo [21] and Yamanishi [22] benchmark datasets are publicly available from their original sources, as described in the corresponding original publications.

## Supplementary Materials

The following supporting information can be downloaded at: https://www.mdpi.com/article/10.3390/biom16060908/s1, Table S1: Paired Wilcoxon signed-rank test results between NAFF-DTI and the strongest baseline for each dataset and metric; Table S2: Performance comparison of NAFF-DTI under different candidate negative settings; Table S3: Source-level ablation results of NAFF-DTI on the IDTI and Luo datasets; Table S4: Top-K performance of NAFF-DTI under different cold-start settings on the Luo dataset; Table S5: Low-frequency group performance of NAFF-DTI and *w*/*o*-adapt under the Luo long-tail settings.
