# Supplementary material for: A Node-Adaptive Feature Fusion Network for Drug–Target Interaction Prediction Based on Multi-View Graphs
Source: Biomolecules. 2026 Jun 18;16(6):908. doi: 10.3390/biom16060908 (PMC13297179; doi:10.3390/biom16060908)
Supplement: Supplementary file 1 [file biomolecules-16-00908-s001.zip › biomolecules-4354727-supplementary.pdf]

Supplementary Materials

S1. Statistical significance analysis against the strongest baselines

To further assess whether the performance improvements of NAFF-DTI over the most competitive baseline methods were statistically supported, paired Wilcoxon signed-rank tests were conducted based on the five-fold test results. For each dataset and each evaluation metric, the strongest baseline was defined as the non-NAFF-DTI method with the highest average performance under the same evaluation protocol. One-sided paired tests were performed to examine whether NAFF-DTI achieved higher performance than the corresponding strongest baseline. The significance level was set to 0.05.

Table S1. Paired Wilcoxon signed-rank test results between NAFF-DTI and the strongest baseline for each dataset and metric.

Dataset	Metric	Comparison	NAFF-DTI	Baseline	Mean difference	Wilcoxon statistic	p-value	Significance
IDTI	AUC	NAFF-DTI vs NASNet-DTI	0.9394 ± 0.0114	0.9018 ± 0.0036	0.0376	15	0.03125	*
IDTI	AUPR	NAFF-DTI vs NASNet-DTI	0.9528 ± 0.0094	0.9152 ± 0.0025	0.0376	15	0.03125	*
Luo	AUC	NAFF-DTI vs DDGAE	0.9460 ± 0.0094	0.8905 ± 0.0275	0.0555	15	0.03125	*
Luo	AUPR	NAFF-DTI vs MKDTI	0.9549 ± 0.0089	0.9111 ± 0.0105	0.0439	15	0.03125	*
GPCR	AUC	NAFF-DTI vs NASNet-DTI	0.9572 ± 0.0151	0.9138 ± 0.0115	0.0439	14	0.06250	ns
GPCR	AUPR	NAFF-DTI vs MKDTI	0.9620 ± 0.0133	0.9248 ± 0.0115	0.0372	14	0.06250	ns
Enzyme	AUC	NAFF-DTI vs DDGAE	0.9928 ± 0.0031	0.9733 ± 0.0060	0.0196	15	0.03125	*
Enzyme	AUPR	NAFF-DTI vs DDGAE	0.9941 ± 0.0025	0.9783 ± 0.0038	0.0158	15	0.03125	*
IC	AUC	NAFF-DTI vs NASNet-DTI	0.9889 ± 0.0042	0.9707 ± 0.0067	0.0182	15	0.03125	*
IC	AUPR	NAFF-DTI vs NASNet-DTI	0.9914 ± 0.0029	0.9756 ± 0.0057	0.0158	15	0.03125	*

* indicates $p < 0.05$, and ns indicates not significant. The p-values were calculated using one-sided paired Wilcoxon signed-rank tests based on the five-fold results.

As shown in Table S1, NAFF-DTI achieved statistically significant improvements in eight out of ten dataset–metric comparisons. The improvements were significant for both AUC and AUPR on the IDTI, Luo, Enzyme, and IC datasets. On the GPCR dataset, NAFF-DTI obtained higher mean AUC and AUPR than the corresponding strongest baselines, but the p-values did

not reach the 0.05 significance level. This may be related to the relatively small scale of the GPCR dataset. After five-fold splitting, each test fold contains fewer interaction samples, making the statistical test more sensitive to fold-level fluctuations.

S2. Evaluation under different candidate negative settings

To evaluate whether the reported performance was affected by the test negative sampling ratio, we additionally compared the original 1:1 setting with two more imbalanced candidate negative settings, namely the 1:10 setting and the all-candidate setting, on the IDTI and Luo datasets. In the 1:10 setting, each held-out positive interaction was evaluated together with ten randomly sampled unobserved drug–target pairs. In the all-candidate setting, each held-out positive interaction was evaluated together with all available unobserved drug–target pairs after excluding known positive pairs in the training and test sets. The training procedure and validation-based model selection remained unchanged across all settings.

Compared with the 1:1 setting, AUPR decreased markedly under the 1:10 and all-candidate settings because the positive ratio in the candidate set was substantially reduced. This decrease was especially pronounced under the all-candidate setting, where the test candidate space became highly imbalanced. Nevertheless, NAFF-DTI maintained relatively high AUC values on both datasets, indicating that the model still preserved effective overall ranking ability under more challenging and realistic candidate spaces.

Table S2. Performance comparison of NAFF-DTI under different candidate negative settings.

Setting/Metric	IDTI	Luo
1:1 AUC	0.9394±0.0114	0.9460±0.0094
1:1 AUPR	0.9528±0.0094	0.9549±0.0089
1:10 AUC	0.8947±0.0146	0.9238±0.0169
1:10 AUPR	0.6603±0.0401	0.7211±0.0550
All-candidate AUC	0.8969±0.0060	0.9162±0.0140
All-candidate AUPR	0.0705±0.0055	0.2107±0.0491

S3. Source-level ablation analysis of drug and target similarity views

To further evaluate the contribution of different auxiliary similarity sources, source-level ablation experiments were conducted on the IDTI and Luo datasets. Specifically, the target similarity view and the drug similarity view were separately removed by replacing the corresponding similarity graph with an identity matrix after data preprocessing, while keeping

the training, validation, and test splits unchanged. As shown in Table S3, removing either the target similarity view or the drug similarity view led to a slight decrease in AUC and AUPR compared with the full NAFF-DTI model. These results indicate that both drug and target similarity views provide useful auxiliary information, while NAFF-DTI can still maintain competitive performance through the interaction view and the remaining similarity source.

Table S3. Source-level ablation results of NAFF-DTI on the IDTI and Luo datasets.

Model	IDTI AUC	IDTI AUPR	Luo AUC	Luo AUPR
w/o-target-sim	0.9355±0.0077	0.9491±0.0052	0.9376±0.0085	0.9497±0.0074
w/o-drug-sim	0.9331±0.0077	0.9479±0.0059	0.9325±0.0179	0.9448±0.0162
NAFF-DTI	0.9394±0.0114	0.9528±0.0094	0.9460±0.0094	0.9549±0.0089

S4. Numerical summary of cold-start performance under different settings

Figure 4 in the main text visualizes the nonzero cold-start results for clearer comparison. As a numerical summary, Table S4 reports the Hit@10, Recall@10, and NDCG@10 values of NAFF-DTI under protein-, drug-, and both-side cold-start settings on the Luo dataset. The Both Cold-start setting yielded zero values for all three metrics across repeated runs and was therefore not plotted in Figure 4.

Table S4. Top-K performance of NAFF-DTI under different cold-start settings on the Luo dataset.

Cold-start setting	Hit@10	Recall@10	NDCG@10
Protein cold-start	0.3000 ± 0.1061	0.1113 ± 0.0277	0.1297 ± 0.0453
Drug cold-start	0.3137 ± 0.0618	0.1639 ± 0.0511	0.1280 ± 0.0636
Both cold-start	0	0	0

S5. Low-frequency long-tail performance comparison between NAFF-DTI and *w/o*-adapt

To further examine whether the node-level adaptive fusion mechanism contributes to long-tail prediction, we compared the full NAFF-DTI model with its *w/o*-adapt variant under the same Luo long-tail settings. Since Figure 5 in the main text reports the performance of NAFF-DTI across low-, mid-, and high-frequency groups, this supplementary analysis focuses on the low-frequency groups, where long-tail bias is most evident. As shown in Table S5, NAFF-DTI consistently achieved higher Hit@10, Recall@10, and NDCG@10 than *w/o*-adapt in the protein-, drug-, and both-side long-tail settings. These results suggest that the adaptive fusion mechanism improves prediction for low-frequency nodes and partially mitigates long-tail bias, although the long-tail problem is not fully eliminated.

Table S5. Low-frequency group performance of NAFF-DTI and *w/o*-adapt under the Luo long-tail settings.

**Long-tail setting**	**Model**	**Low Hit@10**	**Low Recall@10**	**Low NDCG@10**
Protein long-tail	*w/o*-adapt	0.1600 ± 0.0822	0.1450 ± 0.0798	0.0771 ± 0.0444
Protein long-tail	NAFF-DTI	0.2500 ± 0.0935	0.2350 ± 0.0742	0.1555 ± 0.0549
Drug long-tail	*w/o*-adapt	0.3600 ± 0.0548	0.3550 ± 0.0570	0.2309 ± 0.0522
Drug long-tail	NAFF-DTI	0.4900 ± 0.0962	0.4650 ± 0.0962	0.3283 ± 0.0639
Both long-tail	*w/o*-adapt	0.1200 ± 0.0837	0.1200 ± 0.0837	0.0566 ± 0.0396
Both long-tail	NAFF-DTI	0.1600 ± 0.0822	0.1600 ± 0.0822	0.0747 ± 0.0304
